# Biophysical Evaluation and In Vitro Controlled Release of Two Isomeric Adamantane Phenylalkylamines with Antiproliferative/Anticancer and Analgesic Activity

**DOI:** 10.3390/molecules27010007

**Published:** 2021-12-21

**Authors:** Marilena Vlachou, Angeliki-Sofia Foscolos, Angeliki Siamidi, Angeliki Syriopoulou, Nikitas Georgiou, Aikaterini Dedeloudi, Antonis D. Tsiailanis, Andreas G. Tzakos, Thomas Mavromoustakos, Ioannis P. Papanastasiou

**Affiliations:** 1 Division of Pharmaceutical Technology, Department of Pharmacy, School of Health Sciences, National and Kapodistrian University of Athens, Panepistimioupoli-Zografou, 157 84 Athens, Greece; asiamidi@pharm.uoa.gr (A.S.); dedeloud@pharm.uoa.gr (A.D.); 2 Division of Pharmaceutical Chemistry, Department of Pharmacy, School of Health Sciences, National and Kapodistrian University of Athens, Panepistimioupoli-Zografou, 157 84 Athens, Greece; asfoscolou@pharm.uoa.gr; 3 Department of Organic Chemistry, Faculty of Chemistry, National and Kapodistrian University of Athens, Panepistimioupoli-Zografou, 157 71 Athens, Greece; syriopouloua@chem.uoa.gr (A.S.); nikitasgalleti93@hotmail.com (N.G.); tmavrom@chem.uoa.gr (T.M.); 4 Section of Organic Chemistry and Biochemistry, Department of Chemistry, University of Ioannina, 451 10 Ioannina, Greece; antonis.tsiailanis@gmail.com (A.D.T.); atzakos@uoi.gr (A.G.T.); 5 Institute of Materials Science and Computing, University Research Center of Ioannina (URCI), 451 10 Ioannina, Greece

**Keywords:** adamantane phenylalkylamines, 1D and 2D NMR spectroscopy, computational analysis, enzymatic stability, controlled release studies

## Abstract

The aqueous dissolution profile of the isomeric synthetic adamantane phenylalkylamine hydrochlorides **I** and **II** was probed. These adducts have shown significant antiproliferative/anticancer activity associated with an analgesic profile against neuropathic pain. They are both devoid of toxic effects and show appreciable enzymatic human plasma stability. The structures of these two compounds have been elucidated using 2D NMR experiments, which were used to study their predominant conformations. Compound **II**’s scaffold appeared more flexible, as shown by the NOE spatial interactions between the alkyl bridge chain, the aromatic rings, and the adamantane nucleus. Conversely, compound **I** appeared very rigid, as it did not share significant NOEs between the aforementioned structural segments. MD simulations confirmed the NOE results. The aqueous dissolution profile of both molecules fits well with their minimum energy conformers’ features, which stem from the NOE data; this was nicely demonstrated, especially in the case of compound **II**.

## 1. Introduction

The advent of the current global pandemic, COVID-19, has not only highlighted the need for new active pharmaceutical ingredients (APIs), but it has also given credence to the development of novel and efficient drug delivery systems. The application of effective chemical entities coupled with advances in materials science provides a glimmer of hope for solving the most prevalent global health challenges. Moreover, the oral route is the primary and most convenient method of administering drugs; it allows self-administration and is associated with good patient compliance compared with the other options [1,2,3]. Consequently, oral dosage forms of drug candidates remain the main target in research and marketing. Unfortunately, despite the advancements in delivery systems and progress in state-of-the-art of drug design and development, solubility and stability remain the Achilles heel of the APIs [4].

**Figure 1:**
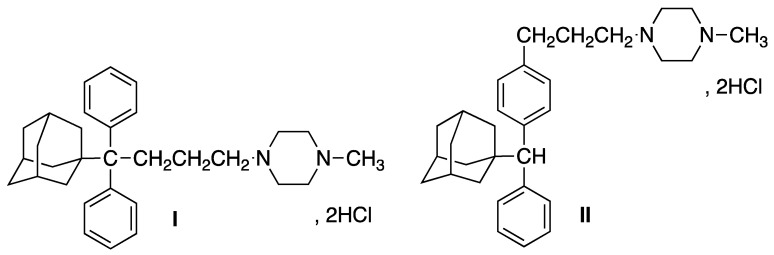
Chemical structures of compounds **I** and **II**.

In this work, we focus on the oral modified release of two potential APIs, the isomeric 4-[4,4-diphenyl-4-[(1-adamantyl)butyl)]-1-methylpiperazine (**I**) and 4-{3-[4-[*α*-(1-adamantyl)phenylmethyl]phenyl]propyl}-1-methylpiperazine (**II**) (Figure 1), the synthesis and pharmacology of which we have reported [5,6], in the context of our ongoing effort to exploit adamantane’s role in antiproliferative activity [7,8,9,10]. 

The adamantane phenylalkylamine hydrochlorides **I** and **II** were found to exhibit a significant sigma-receptor (σR) binding affinity and antiproliferative/ anticancer activity, associated with an analgesic profile against neuropathic pain. Anavex Life Sciences Corp. Pharma has further explored these important properties by subjecting derivative **II** (AV1066) to preclinical phase studies against both neuropathic and visceral pain [11]. Sigma receptors (σRs) represent an enigmatic class of proteins [12] involved in a plethora of biochemical pathways [13,14] and are accommodated by a broad structural diversity of ligands which exhibit a variety of pharmacological activities, such as anticancer [15,16], neuroprotective [17,18] and even coronavirocidal activities [19,20,21].

The important pharmacological properties of these two adamantane adducts prompted us to assess their enzymatic stability. Both analogues were relatively stable for 24 h of incubation in human plasma at 37 °C. Furthermore, the pharmacokinetics and toxicity characteristics of the two compounds were explored and predicted to be neither hepatotoxic nor active for all the associated toxicological pathways according to the ProTox-II platform.

Thus, the next step in our studies was to conduct 2D NMR molecular structure elucidation experiments of compounds **I** and **II**, which were additionally confirmed by MD simulations, in an attempt to decipher whether their matrix tablets formulations could transduce them into orally administered APIs. Moreover, the human plasma stability of the corresponding adducts and their pharmacokinetic properties and toxicities were evaluated.

The in vitro release profiles of adducts **I** and **II** were monitored in the gastric and intestinal pH environment. At pH 1.2, compound **I**, in its hydrochloride form, is endowed with high hydrophilicity, but at neutral pH, where it is transformed to its free base, it becomes lipophilic (logP = 6.073). The hydrochloride salt of compound **II** shows a different profile. Specifically, at pH 1.2 it is sparingly soluble, but at neutral pH, it is equally lipophilic (logP = 6.071). 

Taking into account these characteristics, we designed and prepared oral dosage forms using the appropriate matrix systems. Apart from the bioactive substances **I** and **II**, the formulations included the biopolymers hydroxypropylmethycellulose (HPMC), poly(ethylene oxide) (PEO), Eudragit, and the marine biopolymer ulvan. In order to modulate the aqueous dissolution profile of compounds **I** and **II** in simulated media along the entire gastrointestinal tract, various ratios of these formulants were used.

Matrix-based modified drug delivery systems are complex networks of either hydrophilic or hydrophobic polymers homogeneously mixed with active substances. The release rate of the active substances from matrix-type systems is strongly associated with polymer-related factors, such as the type, the combination, the proportions and the particle properties of the polymers, but also with drug-related factors, such as drug solubility, molecular weight, size, particle size and shape.

As far as drug release is concerned, solubility in aqueous media is associated with properties of the drug substances, such as the physicochemical nature of the functional ligands, the stereochemical configuration and polymorphism. When active substances are characterized by high solubility, the release rate is rapid, while substances of low solubility present a retarded release rate. In particular, in matrix-type systems, the release rate is enhanced by using a variety of polymers which contribute to the solubility potential of the active substances. Thus, the release kinetics for soluble substances are conducted by diffusion and erosion of the polymers, whilst for insoluble substances, osmosis and erosion phenomena of the polymers are dominant [22,23,24]. Moreover, the utilization of pH-controlled aqueous media contributes to the creation of more stable and uniform dissolution conditions, mainly when the active substance is characterized by low solubility [25,26]. Polymers such as ulvan and Eudragit are pH-dependent soluble polymers, releasing the drug at a lesser rate in environments with an acidic pH (1.2).

A number of considerable aspects influence and determine drug release from matrix-type modified drug delivery systems, including the rate of aqueous medium penetration into the polymeric matrix, which results in hydration, gelation and swelling of the polymers and affects the rate of polymeric matrix erosion [27].

## 2. Results and Discussion

### 2.1. Post Compression Parameters

Thickness Test: In all cases, tablets of 10 mm diameter and thickness (2 ± 0.01 mm) were produced. 

Hardness Test: The surface hardness of each tablet is expressed in N and is in the range of 98.20–115.50.

The physical properties of the new formulations were considered, in all cases, acceptable.

### 2.2. NMR

**Figure 2:**
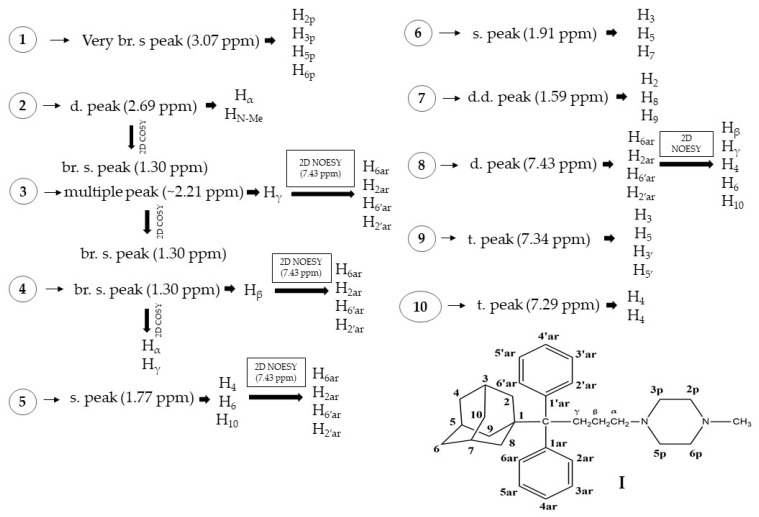
Molecular structure of compound **I** and the strategy followed for the elucidation of its structure.

The structure of compound **I** has been identified by 2D NMR ^1^H-^1^H COSY and 2D ^1^H-^1^H NOESY experiments. The strategy used for the structural identification of its protons is presented in Figure 2.

As a starting point for the assignment of the protons, the very broad single peak at 3.07 ppm was used, which corresponds to H_2p_, H_3p_, H_5p_ and H_6p_, as these protons are highly deshielded by the two vicinal nitrogens. Then, at 2.69 ppm, a doublet was observed which was integrated to 5 protons which correspond to H_α_ and H_N-Me_, as these protons are deshielded by the vicinal nitrogens. Using 2D ^1^H-^1^H COSY, H_γ_ has been shown to resonate at 2.21 ppm. The 2D ^1^H-^1^H NOESY experiment demonstrated the NOE effect between H_c_ and the hydrogens corresponding to the double peak at 7.43 ppm, which were assigned to H_6ar_, H_2ar_, H_6′ar_ and H_2′ar_. The next step was the identification of the broad single peak at 1.30 ppm, which showed an NOE effect with the hydrogens H_6ar_, H_2ar_, H_6′ar_ and H_2′ar_ (7.43 ppm). This peak corresponds to H_β_, which is confirmed by the 2D ^1^H-^1^H COSY spectrum presented in Supplementary Material Figure S3.

The hydrogens, which resonate at the 1.77 ppm peak, belong to the adamantane ring and show a NOE effect (Figure S2) with hydrogens H_6ar_, H_2ar_, H_6′ar_ and H_2′ar_ (7.43 ppm), signifying their spatial proximity. Hence, the peak at 1.77 ppm corresponds to H_4_, H_6_ and H_10_, which are in spatial proximity to H_6ar_, H_2ar_, H_6′ar_ and H_2′ar_.

The peaks at 1.91 and 1.59 ppm correspond to H_3_, H_5_, H_7_ and H_2_, H_8_, H_9_, respectively. Lastly, in the aromatic area (6.5–8.5 ppm), the peak at 7.43 ppm corresponds to H_6ar_, H_2ar_, H_6′ar_ and H_2′ar_, which is verified by the NOE effect (Figure S4) shown between these protons with H_4_, H_6_ and H_10_. The peak resonating at 7.29 ppm corresponds to H_4ar_, H_4ar’_, and at 7.34 ppm, it corresponds to H_3_, H_5_, H_3′_ and H_5′_ (vide Figure S1). The aforementioned results are summarized in Table S1 (vide Supplementary Materials).

^1^H NMR, 2D ^1^H-^1^H COSY and 2D ^1^H-^1^H NOESY experiments were also conducted for compound **II** following the same strategy (Figure S5).

The protons of the six-membered heterocyclic ring, H_2p_, H_3p_, H_5p_ and H_6p_, are the most deshielded, as they are in the vicinity of the two nitrogens and resonate as broad multiple peaks at 3.61 ppm. The protons H_N-Me_, H_α,_ and H_γ_ resonate at 2.99 ppm, 2.70 ppm and 3.21 ppm, respectively. The correlation detected in 2D COSY experiments (Figure S6) between H_α_ and H_γ_ and the peak at 2.05 ppm is assigned to H_β_, as it shows a bond correlation with the H_α_ and H_γ_ protons. 

The protons of the adamantane ring resonate in the area of 1.37–1.98 ppm. The single peak at 1.86 ppm corresponds to H_3_, H_5_ and H_7_ of the adamantane nucleus. The peak at 1.62 ppm corresponds to H_4_, H_6_, H_10_, H_2eq_, H_8eq_ and H_9eq_, and at 1.53 ppm, it corresponds to H_2ax_, H_8ax_ and H_9ax_. Figure S2 gives an overall picture of all the protons of compound **II**. 2D ^1^H-^1^H NOESY experiments were also conducted on compound **II**; it was found that the protons of the phenyl groups are in spatial proximity with the protons of the adamantane ring and with the protons of carbons C_α_, C_β,_ and C_γ_. Figure S7 presents the 2D NOESY spectrum, and the results of the analysis are shown in Table S2.

### 2.3. Molecular Dynamic Results

**Figure 3:**
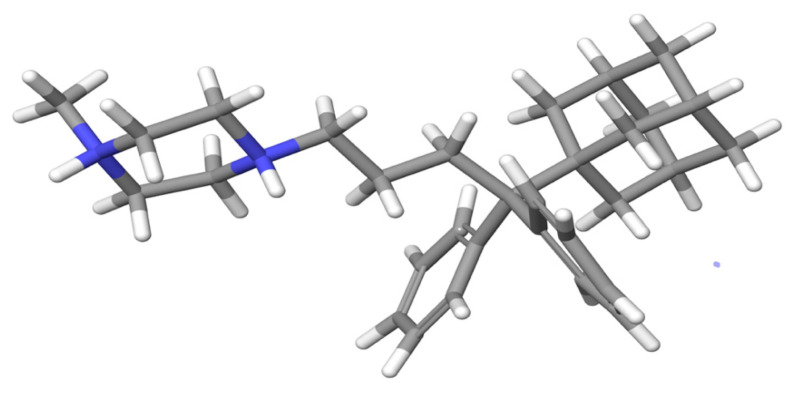
The predominant (85%) conformation after the MD simulation of compound **I**.

Over the course of 500 ns, the MD simulations indicate that the most predominant conformation of compound **I** is as presented in Figure 3; compound **I** has shown similar conformations at 85% of the simulation time. The depicted conformation explains the spatial correlations found in the 2D NOESY spectrum.

**Figure 4:**
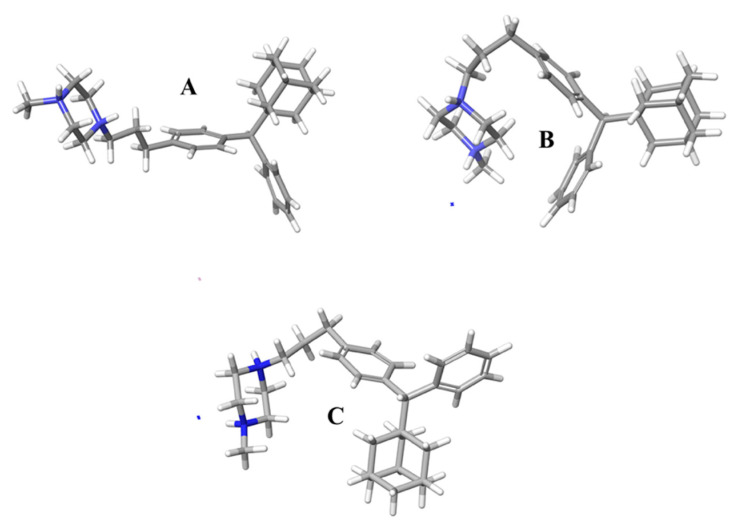
Conformational analysis of compound **II** after the MD simulation time. ((**A**–**C**) means different conformations of compound **II**, derived during the trajectory simulation time).

On the other hand, compound **II** presented structural deviations over time, as illustrated in the prevailing adopted conformations A, B, and C over 500 ns (Figure 4). The structures below indicate the greater flexibility of compound **II**. The depicted conformations explain the plethora of the spatial correlations observed in the 2D NOESY spectrum.

### 2.4. In Vitro Dissolution Studies

**Figure 5:**
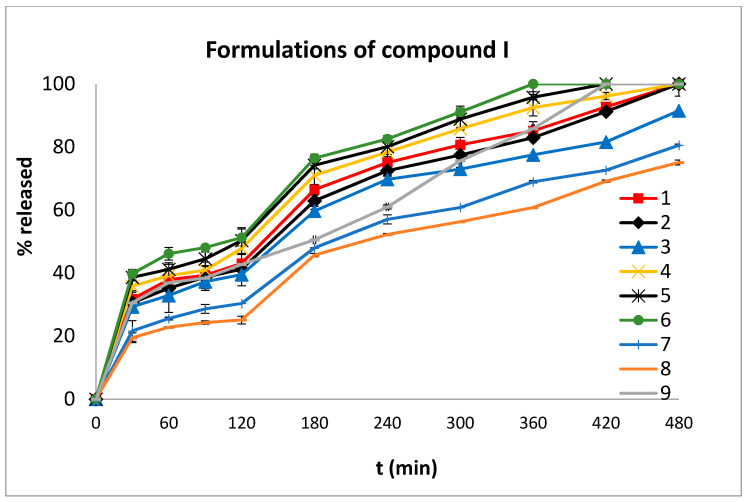
In vitro % release of compound **I** versus time from tablet formulations 1–9 at pH 1.2 (0–120 min) and at pH 6.8 (120–480 min). The results denote the mean value ± SD (*n* = 3).

**Figure 6:**
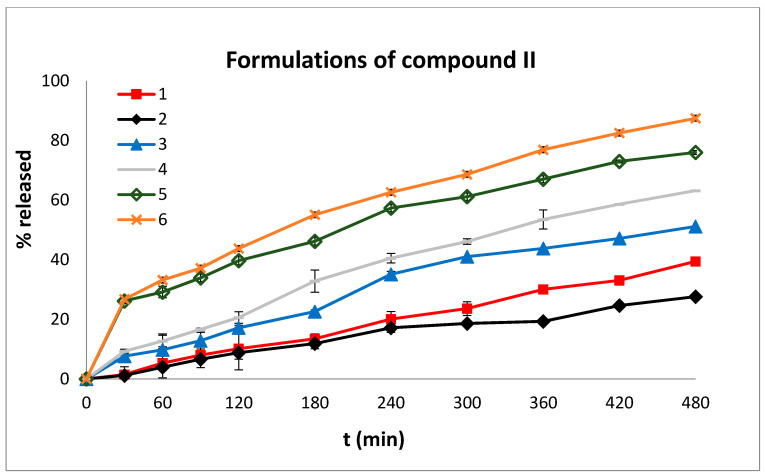
In vitro % release of compound **II** versus time from tablet formulations 1–6 at pH 1.2 (0–120 min) and at pH 6.8 (120–480 min). The results denote the mean value ± SD (*n* = 3).

The dissolution profiles of both compounds **I** and **II** are shown in Figure 5 and Figure 6, respectively.

The kinetic release data regarding the developed formulations of compounds **I** and **II** are reported in Table 1. The terms t_20%_, t_50%_ and t_90%_ refer to the time when 20%, 50%, and 90%, respectively, of the dissolution process has been achieved. MDT refers to the mean dissolution time, while D.E.% refers to % dissolution efficiency and n denotes the release kinetics according to the power law.

It is apparent from the data presented in Figure 5 and Figure 6 that the % release of compound **I** from formulations 1–9 was higher than that of compound **II** from formulations 1–6 at both pHs (1.2 and 6.8). Specifically, the % release of compound **I** from the formulations ranged from 25.08% (formulation 8) to 51.25% (formulation 6) at the acidic pH (120 min) and from 75.08% (formulation 8) to 100% (formulations 1,2, 4–7) at the neutral pH (480 min). The % release of compound **II** from the formulations ranged from 8.83% (formulation 2) to 43.75% (formulation 6) at the acidic pH (120 min) and from 27.61% (formulation 2) to 87.46% (formulation 6) at the neutral pH (480 min). 

The release of compound **I** (Figure 6) from the formulations that contained poly(ethylene oxide), PEO (1–3), in a larger amount (44%), was high (≈40%; pH 1.2). An enhanced release of compound **I**, from the same formulations, was also noticed at pH 6.8. PEO is the most widely used excipient in controlled release matrix tablets with unique swelling and erosion properties which can be utilized in modulating drug release profiles [28].

**Figure 7:**
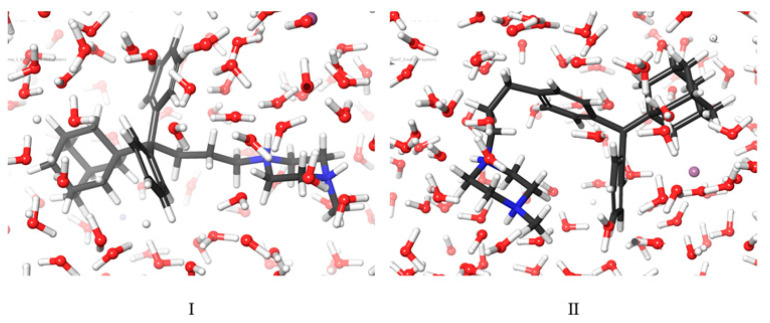
Snapshots of compounds **I** & **II** obtained during the MD simulations.

The increase of % Eudragit L100-55 (25% in formulation 4) led to an increase in the total % release of compound **I** (DE% = 69.83) when compared to formulations 1–3 (DE% = 66,09, 64.04 and 59.89, respectively, Figure 7). Eudragit L100-55 is used to lower drug release at acidic pH values and promote its release at neutral pH values. However, in this case, when Eudragit L100-55 was used, a higher release of the active substance was observed in time-points which correspond to acidic conditions. This could be attributed to the use of a large amount of Eudragit L100-55 in the matrix tablet, resulting in augmented water penetration into the matrix [29,30].

The tablets containing ulvan (12.5% and 25%) (Formulations 5 and 6 respectively) (Figure 5) showed a relatively higher drug release (5: 50.30%, at 120 min and 100% at 420 min; 6: 51.25%, at 120 min and 100% at 420 min) than all the other formulations. This is possibly due to the unusual chemical composition of ulvan, which is highly sulphated and contains rhamnose 3-sulphate, xylose, xylose 2-sulphate, glucuronic acid and iduronic acid residues. Thus, in the acidic environment, a number of ulvan’s functionalities remained ionized, facilitating the dissolution of compound **I**. It is noteworthy that this dissolution-enhancing effect of ulvan was maintained at pH 6.8. Both the sulfate and the carboxylate groups of ulvan are ionized at this pH, thus facilitating drug release. Apart from the pH and the functionalities of ulvan, which affect the drug dissolution, the amorphous shape and size of ulvan also contribute to the enhanced drug release. Moreover, ulvan’s particles have a sponge-like shape with large cavities through which the molecules penetrate faster and deeper [31].

The release of compound **I** (Figure 5) from formulations 7 and 8 (both contain 44% hydroxypropylmethylcellulose (HPMC)) showed a decrease in relation to all the other formulations (which contain 27.5% and 24% HPMC) (D.E.% 7: 50.35, 8: 46.10, while all other formulations >59). This is in agreement with previously published data, as in swellable matrices containing HPMC K15M, the release of the drug is affected by one or more processes, e.g., anomalous transport (non-Fickian), which refers to the coupling of the Fickian diffusion and polymer matrix relaxation (*n* = 0.57 and 0.59 for formulations 7 and 8, respectively) [32,33].

The release of compound **II** (Figure 6) from the formulations 1 to 6 showed an increase as the % of Eudragit L100-55 and sodium alginate was increased. As mentioned previously, the use of Eudragit L100-55 contributes to augmented water penetration of the matrix tablets, resulting in its erosion and, therefore, the facile dissolution of the API. More particularly, formulations 5 and 6, which contained the larger amounts of Eudragit L100-55 (47.5% and 56%, respectively) showed *n* values of 0.43 and 0.44, respectively, which correspond to a Fickian diffusion release profile. Fickian diffusion refers to the solute transport process in which the polymer relaxation time is much greater than the characteristic solvent diffusion time.

It is plausible that the release of compound **II**, irrespective of the qualitative and quantitative characteristics of the excipients present in its matrix tablets, is generally lower than that of analogue **I**, because in the predominant conformations of the former (A, B and C, Figure 4) the entrapment of large amounts of water molecules between the two phenyl rings is sterically prevented, and as a result, the formation of H-bonds between water and the π-system of the phenyl rings is not very probable. It has been well documented that both the hydrogen atoms of water pointing toward the π-cloud of benzene form H-bonds leading to aqueous-*π* electron interactions. This explains the partial solubility of benzene in water [34]. Conversely, in the case of compound **I**, the two phenyl rings are in spatial proximity (vide minimum energy conformer, Figure 4), allowing the water molecules to be entrapped between them, leading to effective bilateral aqueous-*π* electron interactions and, hence, to the increased solubility of compound **I** in the dissolution media [35] (Figure 7).

### 2.5. Plasma Stability

**Figure 8:**
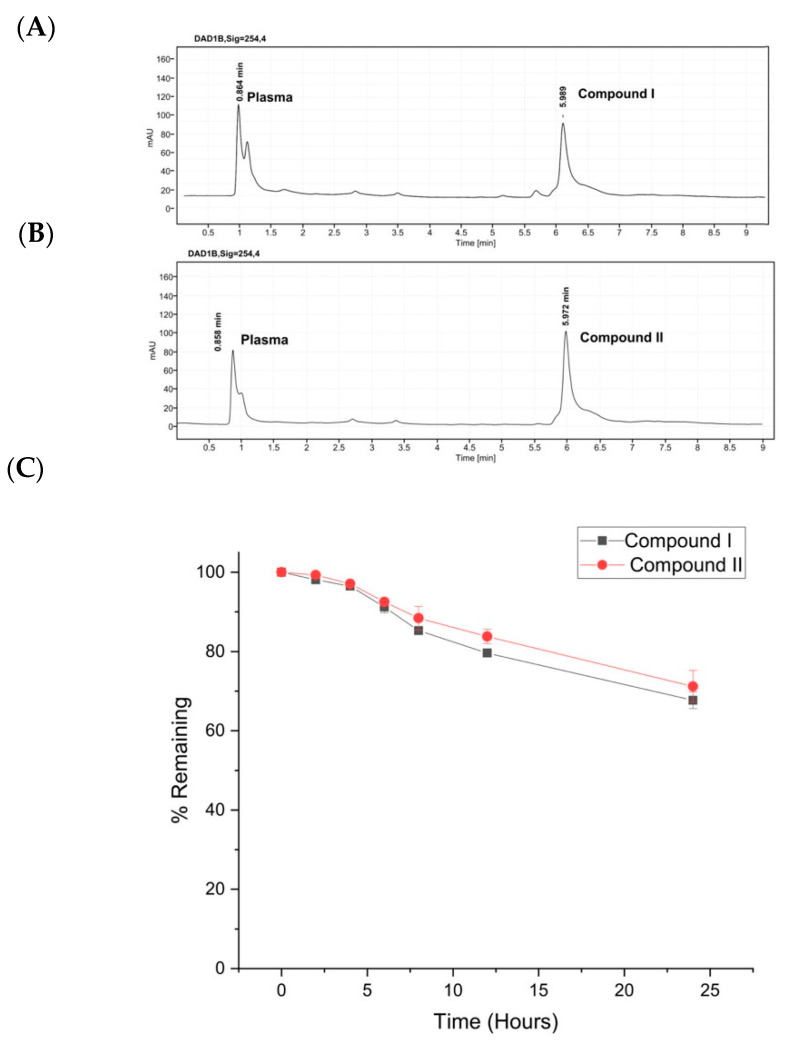
(**A**) Analytical high-performance liquid chromatogram (HPLC) of compound **I** in human plasma at 254 nm. (**B**) Analytical high-performance liquid chromatogram (HPLC) of compound **II** in human plasma at 254 nm. (**C**) Human plasma stability of compound **I** (black color) and compound **II** (red color) after 24 h incubation in human plasma. Experiments were conducted in triplicates.

To evaluate the stability of the compounds, stability studies were performed in human plasma, and the decomposition rate was monitored via HPLC over time (Figure 8). First, we established a new HPLC method in order to determine the retention times of each compound. The degradation rate of each compound after incubation in human plasma for 2, 4, 6, 8, 12, and 24 h is presented in Figure 8. Both analogues were relatively stable for 24 h of incubation in human plasma at 37 °C.

### 2.6. Results of the Pharmacokinetics and Toxicity Properties of the Two Compounds 

In Table 2, the physicochemical parameters of compound **I** and **II** are shown. 

Both compounds **I** and **II** have one violation in Lipinski’s rule of five due to the value of LogP (LogP > 5). Additionally, they have one violation in Veber’s rule, because they have 7 rotatable bonds. Compound **II** is more soluble than compound **I.**

In Table 3, the pharmacokinetic profiles of compound **I** and **II** are shown. 

According to pkCSM, both compounds readily cross the blood-brain barrier, and as a result, they improve the efficacy of drugs whose pharmacological activity is within the brain.

In Table 4, the toxicological results for compound **I** and **II** are shown.

Compound **I** has been classified at toxicity level 4 with an LD_50_ value of 3000 mg/kg, an average similarity of 87.67% and a prediction accuracy of 70.97% using the ProTox-II platform. It has not been predicted to be hepatotoxic, and it is inactive for all toxicological pathways. Compound **II** has been predicted to be toxicity class 5 with LD 50 value of 2560 mg/kg, an average similarity of 77.65% and a prediction accuracy of 69.26%. It has not been predicted to be hepatotoxic, and it is inactive for all toxicological pathways according to the ProTox-II platform [39].

## 3. Materials and Methods

Compounds **I** (MW: 515.61 g/mol) and **II** (MW: 515.61 g/mol) were provided by colleagues in the Pharmaceutical Chemistry Department. Hydroxypropylmethycellulose (HPMC K100M) and poly(ethylene oxide) (PEO) (MW: 7 × 10^6^ g/mol) were obtained from Sigma-Aldrich (Steinheim, Germany). Eudragit L100-55 was purchased from Rohm GmbH Pharma Polymers (Darmstadt, Germany), and ulvan (Cat. No. YU11689) was bought from Carbosynth^®^ Ltd. (Berkshire, UK). Alginic acid sodium salt (low viscosity) was supplied by Alfa Aesar GmbH & Co KG (Karlsruhe, Germany), and magnesium stearate was purchased from Riedel-De Haen (Hannover, Germany). Deuterium oxide 99.90% D (D214F) was purchased from Eurisotop via Fluorochem (Hadfield, UK). All chemicals were of reagent grade and used in this study without further purification.

### 3.1. Formulation of Compounds **I** and **II** into Modified Release Tablets

The formulations of compounds **I** and **II** were prepared using a variety of polymeric excipients into matrix-type tablet forms (vide Table 5 and Table 6). In specific, the APIs (compounds **I** and **II**) and excipients (HPMC, PEO, Eudragit L100-55, ulvan and sodium alginate) were mixed homogenously in a laboratory scale powder blender at 32 rpm for a time duration of 10 min. Subsequently, the lubricant (magnesium stearate) was added to the mixture, and the blending was continued for another 5 min. Finally, the powder mixture was precisely weighed (200 mg), loaded on a 10 mm diameter matrix and directly compressed using a hydraulic press (Maassen type, MP 150). 

### 3.2. Post Compression Parameters

Thickness Test: The thickness of the tablets was measured using a vernier caliper. 

Hardness Test: The hardness of the tablets was determined using an Erweka hardness tester (Erweka, type TBH28). The force applied was equal to breaking the tablet in a diametric compression. The surface hardness of each tablet is expressed in N.

### 3.3. In Vitro Dissolution Studies

The dissolution tests were performed in a tablet dissolution test apparatus USP type II (Pharmatest, Hainerp, Germany). The experiments were carried out in 2 different aqueous media: at pH 1.2, V_max_ = 450 mL for 2 h, and at pH 6.8, V_max_ = 900 mL for 6 more hours, in order to simulate, in vitro, the pH range along the gastrointestinal tract. The temperature of the dissolution medium was maintained at 37 ± 0.5 °C. The tablet was placed in the bottom of a vessel, equipped with paddles, under sink conditions, and the apparatus was operated at 50 rpm. Samples were withdrawn at predetermined time intervals, and the withdrawn volume of the medium was replenished. The withdrawn samples were filtered and analyzed using UV-Vis spectrophotometry (LLG-uniSPEC 2 Spectrophotometer) at λ_max_ = 214 nm for pH 1.2 and at λ_max_ = 210 nm for pH 6.8 for compound **I** and at λ_max_ = 217 nm for pH 1.2 and pH 6.8 for compound **II**. The % dissolution curve versus time was determined according to the calibration curve of the corresponding API.

In order to compare the dissolution profiles, graphs of % drug release versus time were constructed (Figure 5 and Figure 6) and the *D.E.* (%) value was estimated. According to Khan [40], *D.E.* (%) is a useful parameter for the evaluation of dissolution in vitro and is calculated from the following equation:

(1) $$D.E.\;\left(\%\right)=\frac{\int_{t_{1}}^{t_{2}}y\;dt}{y_{100}\left(t_{2}-t_{1}\right)}\times 100$$

where *y* is the percentage of dissolved product and *D.E.* (%) is the area under the dissolution curve between time points *t*_1_ and *t*_2_. They are expressed as a percentage of the curve at maximum dissolution *y*_100_ over the same time period. Dissolution efficiency, which considers the dissolution profile as a whole, was employed to interrelate dissolution with the other variables used in this study.

Furthermore, t_20%_, t_50%_, and t_90%_, as well as the mean dissolution time (*MDT*) values, were estimated. The t_20%_, t_50%_, and t_90%_ values refer to the time where the 20%, 50%, and 90% of the active substance is released. *MDT* is the value used to characterize the drug release rate from a dosage form, and the following is used to derive an estimate of *MDT* from experimental dissolution data [38]:

(2) $$MDT=\frac{ABC}{W_{\infty }}$$

where *W_∞_* is the maximum amount of the drug substance that is dissolved, and *ABC* is the area between the drug dissolution curve and its asymptote.

The in vitro release data were fitted to the Korsmeyer–Peppas equation:

(3) $$\frac{M_{t}}{M_{\infty }}=Kt^{n}$$

where *M_t_* and *M_∞_* denote the absolute cumulative amount of drug released at time *t* and infinite time, respectively, *k* represents the release rate constant and *n* is the diffusion coefficient. This equation is only valid for the first 60% of the fractional release [37]. In the case of cylindrical tablets, *n* ≤ 0.45 corresponds to a Fickian diffusion release (case I diffusional), 0.45 < *n* < 0.89 corresponds to an anomalous transport, and *n* = 0.89 corresponds to zero-order (case II) release kinetics.

### 3.4. Stability of Compounds **I** and **II** in Human Plasma 

In order to examine the stability of the different compounds in human plasma, we followed the assay described by Di et al. [41]. A stock solution of 1 mg/mL of compounds **I** and **II** were prepared by dissolving the appropriate amounts in DMSO. The final working solutions of 50 μM were prepared by further diluting the stock solutions with H_2_O/MeCN (1:1). Human plasma samples were prepared by incubating 10 μL of each compound separately from the prepared stocks with 90 μL human plasma for 2, 4, 6, 8, 12, and 24 h at 37 °C. To terminate the reactions and precipitate the plasma proteins, 300 μL of ice-cold acetonitrile were added to each sample. Samples were then vortex-mixed and centrifuged at 12,000× *g* for 10 min. The supernatants were collected, syringe filtered and transferred to vials for RP-HPLC analysis. Each sample was studied in triplicate, and the concentration of each analogue was calculated by standard curves.

### 3.5. NMR Experiments

The 1D and 2D COSY and NOESY NMR experiments were conducted in a Bruker Avance 600 spectrometer (600 MHz) at 25 °C in D_2_O (99.5%) and water (0.5%). Standard pulse sequences were used and stored in the library of the spectrometer.

### 3.6. Molecular Dynamics Simulations Method

The structures of compounds **I** and **II** were designed with the aid of the 2D Sketcher in Maestro software. After energy minimization, compounds **I** and **II** underwent molecular dynamics (MD) simulations in order to determine their conformational properties in water.

The MD study system was built with SPC/E modeled water around the structure and neutralized with Na^+^ and Cl^-^ ions until the experimental salt concentration of 0.15 M NaCl was reached. The OPLS2005 force field was used to simulate the system, and long-range electrostatics were treated using the particle mesh Ewald (PME) method and a grid spacing of 0.8 Å. Van der Waals and short-range electrostatic interactions were smoothly truncated at 9.0 Å [42,43]. A Nosé–Hoover thermostat kept the temperature constant [44], while the pressure was controlled with the Martyna–Tobias–Klein method [42]. Periodic boundary conditions were applied, and the dimensions of the simulation box were (30.0 Å × 30.0 Å × 30.0 Å). The volume of the box was minimized by reorienting the solutes in the box. The equations of motion were integrated using the multilevel RESPA integrator [45] with an outer time step of 2 fs for bound interactions and unbound interactions within a cutoff of 9 Å. Each system was equilibrated using the standard Desmond protocol [45]. The system was first relaxed with a Brownian dynamics simulation in the NVT ensemble at T = 298 K, with constraints on the solute heavy atoms. MD simulations were performed using the standard Desmond relaxation protocol, which involves gradual heating and reduction of harmonic constraints first for the solvent atoms and later for the solute heavy atoms. Before the start of the production phase, the system was relaxed in the NPT ensemble without any constraints for 1.0 ns. The production phase of the MD simulation was set to 500 ns, which provides a reasonable sample size to discover the predominant conformation during the simulation time. The MD simulations were run on a workstation using the GPU implementation of the MD simulation code.

### 3.7. Methods to Predict Pharmacokinetic and Toxicity of These Compounds

The pharmacokinetic properties and toxicities were determined using the online programs SwissADME, pro-TOX and pkCSM [36,37,46].

## 4. Conclusions

Two lipophilic isomeric adamantane phenylalkylamines (compounds **I** and **II**) with significant antiproliferative/anticancer and analgesic activity and appreciable enzymatic stability were subjected to in vitro controlled release studies. We have shown that the desired oral absorption profile can be achieved when excipients with physicochemical characteristics compatible with the stereoelectronic properties of these molecules are used. The conformational properties of the two compounds justify their aqueous dissolution profile. The 2D NMR experiments have revealed the spatial structural diversities of the two isomeric derivatives, which are responsible for their different aqueous dissolution profiles. Moreover, the MD simulations confirmed the greater flexibility of compound **II** with respect to adduct **I**. Both derivatives are not predicted to be hepatoxic, being inactive in all toxicological pathways.

## Figures and Tables

**Table 1:** Kinetic release data of the developed formulations of compounds **I** and **II**.

	Formulations	MDT	t_20%_	t_50%_	t_90%_	*n*	Mean % D.E.
Compound **I**	**1**	138.89	18	137	398	0.45	66.09
	**2**	145.70	20	144	411	0.44	64.05
	**3**	139.19	21	152	472	0.52	59.89
	**4**	128.36	16	126	338	0.44	69.83
	**5**	120.89	16	119	310	0.19	72.38
	**6**	114.02	15	107	292	0.18	74.44
	**7**	153.95	28	193	*	0.57	50.35
	**8**	157.64	35	220	*	0.59	46.10
	**9**	159.44	19	175	377	0.35	62.53
Compound **II**	**1**	208.21	369	*	*	0.99	19.33
	**2**	189.56	240	*	*	0.76	14.70
	**3**	175.24	150	460	*	0.72	30.19
	**4**	175.36	116	332	*	0.78	36.96
	**5**	135.41	23	201	*	0.43	51.32
	**6**	186.95	23	175	*	0.44	55.78

* The API did not reach 90% release during the dissolution experiment.

**Table 2:** The physicochemical parameters for compound **I** using pkCSM server [36].

Properties		
	Compound I	Compound II
LogP	6.0726	6.0708
Rotable Bonds	7	7
Hydrogen Bond Acceptors	2	2
Hydrogen Bond Donors	0	0
Surface Area	200.919 (Å^2^)	200.919
Water Solubility	−3.776 (log mol.L^−1^)	−4.112

**Table 3:** The predicted pharmacokinetic profiles for compound **I** and **II** using pkCSM server [37].

Properties		
Absorption		
	**Compound I**	**Compound II**
Caco2 Permeability	1.02(>0.90)	0.966(>0.90)
Intestinal Absorption (Human)	100(>30)	97.152(>30)
Skin Permeability	−2.736(<−2.5)	−2.737(<−2.5)
P-Glycoprotein Substrate	Yes	Yes
P-Glycoprotein **I** Inhibitor	Yes	Yes
P-Glycoprotein **II** Inhibitor	Yes	Yes
Distribution		
VDss (Human)	0.299(<0.45)	0.656(>0.45)
Fraction Unbound (Human)	0.193	0.164
BBB Permeability	1.491(>0.3)	1.471(>0.3)
CNS Permeability	−1.552(>−2)	−1.467(>−2)
Metabolism		
CYP2D6 Substrate	No	No
CYP3A4 Substrate	Yes	Yes
CYP1A2 Inhibitior	No	No
CYP2C19 Inhibitior	No	No
CYP2C9 Inhibitior	No	No
CYP2D6 Inhibitior	Yes	Yes
CYP3A4 Inhibitior	No	No
Excretion		
Total Clearance	0.283	0.404
Renal OCT2 Substrate	No	No

**Table 4:** Cross-validation results for compound **I** and **II** in the ProTox-II platform [38].

	Prediction	Probability	Prediction	Probability
	Compound I		Compound II	
Organ Toxicity				
Hepatotoxicity	Inactive	0.94	Inactive	0.93
Toxicity Endpoints				
Mutagenicity	Inactive	0.64	Inactive	0.56
Carcinogenicity	Inactive	0.79	Inactive	0.82
Cytotoxicity	Inactive	0.80	Inactive	0.77
Immunotoxicity	Inactive	0.99	Inactive	0.92
**Toxicological Pathways**				
Aryl hydrocarbon Receptor (AhR)	Inactive	0.94	Inactive	0.94
Andogen Receptor (AR)	Inactive	0.97	Inactive	0.99
Androgen Receptor Ligand Binding Domain (AR-LBD)	Inactive	0.99	Inactive	0.99
Aromatase	Inactive	0.94	Inactive	0.98
Estrogen Receptor Alpha (ER)	Inactive	0.97	Inactive	0.96
Estrogen Receptor Ligand Binding Domain (ER-LBD)	Inactive	0.98	Inactive	0.99
Peroxisome Proliferator Activated Receptor Gamma (PPAR-Gamma)	Inactive	0.99	Inactive	0.99
Nuclear factor (erythroid-derived 2)-like 2/Antioxidant Responsive Element (nrf2/ARE)	Inactive	0.99	Inactive	0.99
Heat Shock Factor Response Element (HSE)	Inactive	0.99	Inactive	0.99
Mitochondrial Membrane Potential (MMP)	Inactive	0.89	Inactive	0.95
Phosphoprotein (Tumor Supressor) p53	Inactive	0.94	Inactive	0.96
ATPase Family AAA Domain-Containing Protein 5 (ATAD5)	Inactive	0.99	Inactive	0.99

**Table 5:** Composition of compound **I** tablet formulations (mg).

Formulation	1	2	3	4	5	6	7	8	9
**Compound I**	10	10	10	10	10	10	10	10	10
**PEO (7 × 10^6^)**	88	88	88	68	68	68	45	45	45
**HPMC K100**	45	45	55	45	45	45	88	88	45
**Eudragit L100-55**	45	10	-	50	50	25	45	45	50
**Sodium Alginate**	10	45	45	25	-	-	10	-	48
**Ulvan**	-	-	-	-	25	50	-	10	-
**Magnesium Stearate**	2	2	2	2	2	2	2	2	2
**Total**	200	200	200	200	200	200	200	200	200

**Table 6:** Composition of compound **II** tablet formulations (mg).

Formulation	1	2	3	4	5	6
**Compound II**	10	10	10	10	10	10
**PEO (7 × 10^6^)**	100	85	70	55	25	10
**HPMC K100**	38	44	50	45	30	30
**Eudragit L100-55**	50	44	38	50	95	102
**Sodium Alginate**	-	15	30	38	38	46
**Magnesium Stearate**	2	2	2	2	2	2
**Total**	200	200	200	200	200	200

## Data Availability

The data presented in this study are available in Supplementary Materials.

## Supplementary Materials

The following are available online, Figure S1. ^1^H NMR spectrum of compound I obtained using 600 MHz Bruker spectrometer at 25 °C in D_2_O; Figure S2. ^1^H NMR spectrum of compound **II** obtained with 600 MHz Bruker spectrometer at ambient temperature in D_2_O; Figure S3. 2D COSY of compound **I** at ambient temperature in D_2_O; Figure S4. 2D NOESY of compound **I** at ambient temperature in D_2_O, Figure S1. Molecular structure of compound **II** and the strategy followed; Figure S2: 2D COSY of compound **II** at ambient temperature in D_2_O; Figure S3: 2D NOESY of compound **II** at ambient temperature in D_2_O; Table S1. ^1^H chemical structure shifts of compound **I** using a combination of 1D and 2D NMR experiments; Table S2. ^1^H NMR chemical shifts of compound II derived from a combination of 1D and 2D homonuclear NMR experiments.

## Sample Availability

Samples of the compounds **I** and **II** are available from the authors.
