# Supplementary material for: Biophysical Evaluation and In Vitro Controlled Release of Two Isomeric Adamantane Phenylalkylamines with Antiproliferative/Anticancer and Analgesic Activity
Source: Molecules. 2021 Dec 21;27(1):7. doi: 10.3390/molecules27010007 (PMC8746252; doi:10.3390/molecules27010007)
Supplement: Supplementary file 1 [file molecules-27-00007-s001.zip › molecules-1500109-supplementary.pdf]

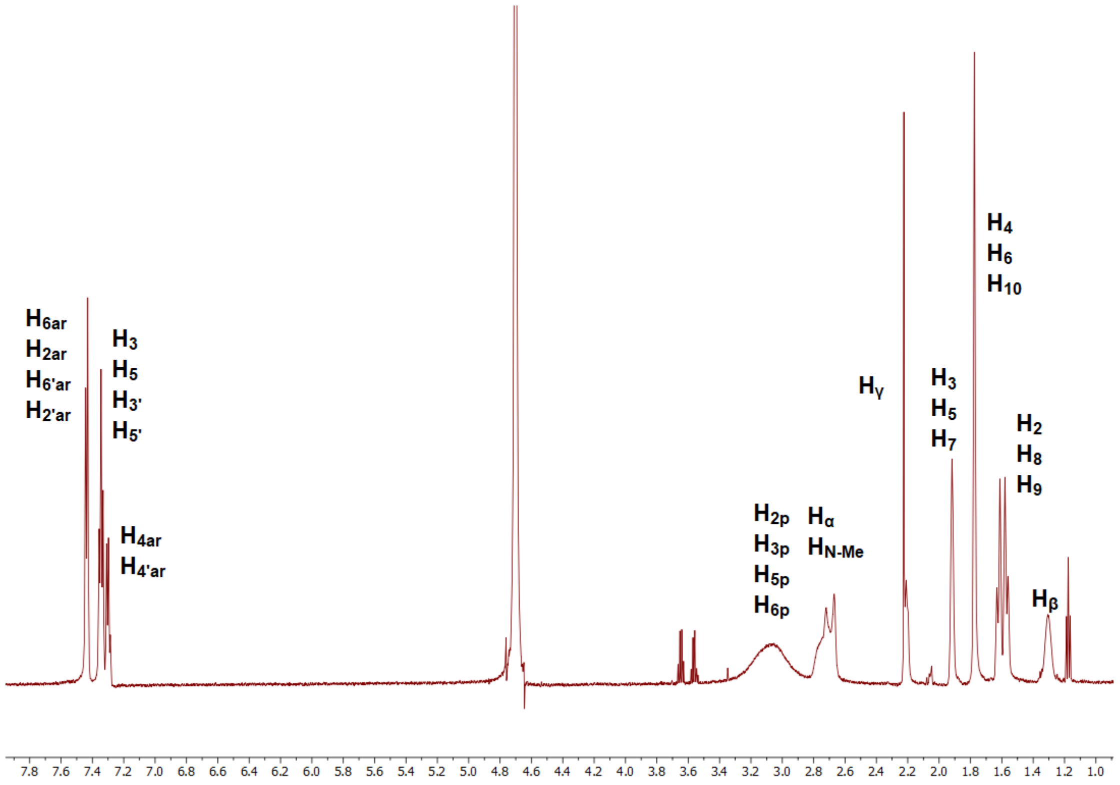

Figure S1. ^{1}H NMR spectrum of compound **I** obtained using 600 MHz Bruker spectrometer at 25 °C in D_2O.

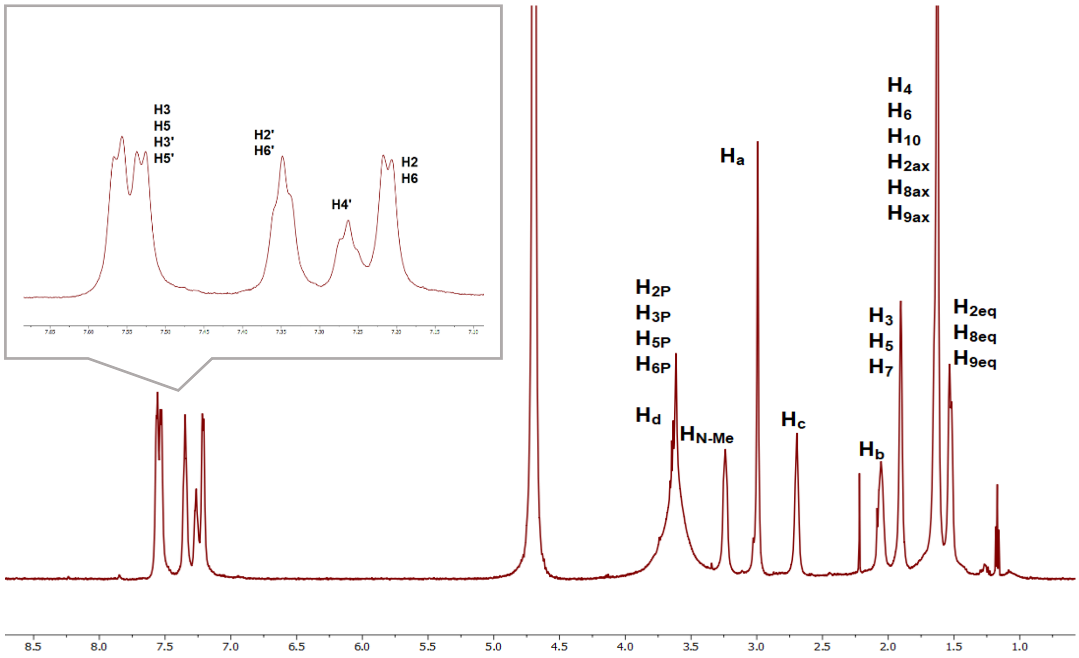

Figure S2. ^{1}H NMR spectrum of compound **II** obtained with 600 MHz Bruker spectrometer at ambient temperature in D_2O.

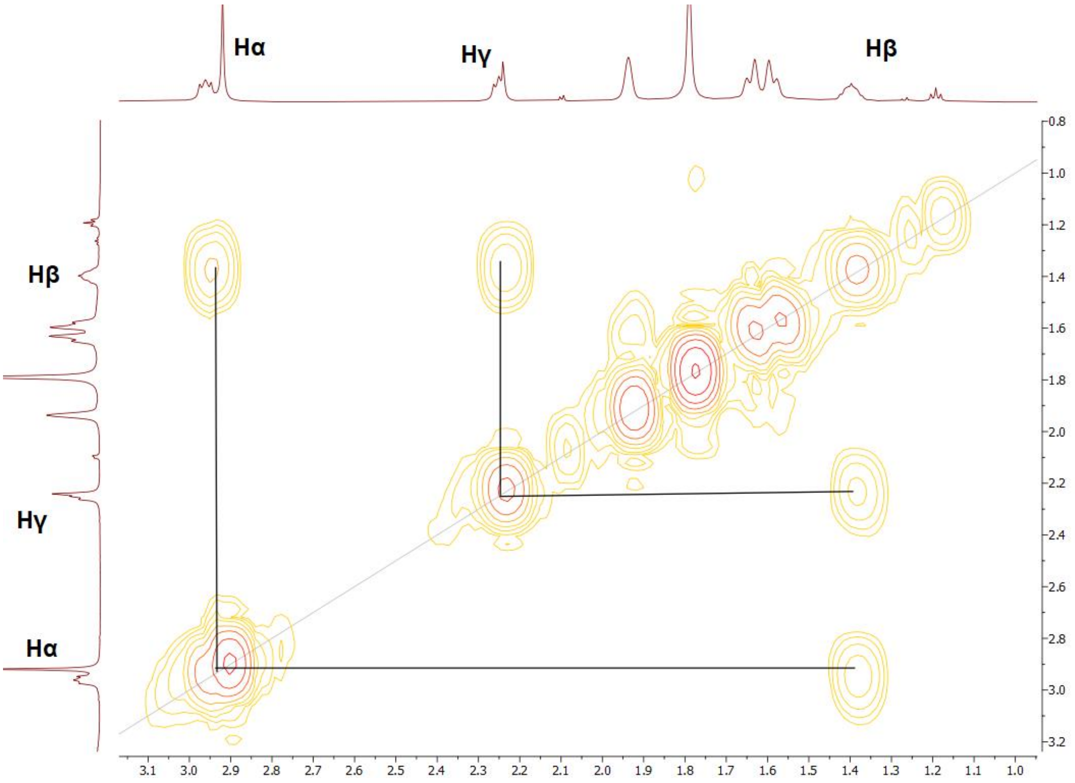

Figure S3. 2D COSY of compound **I** at ambient temperature in D_2O.

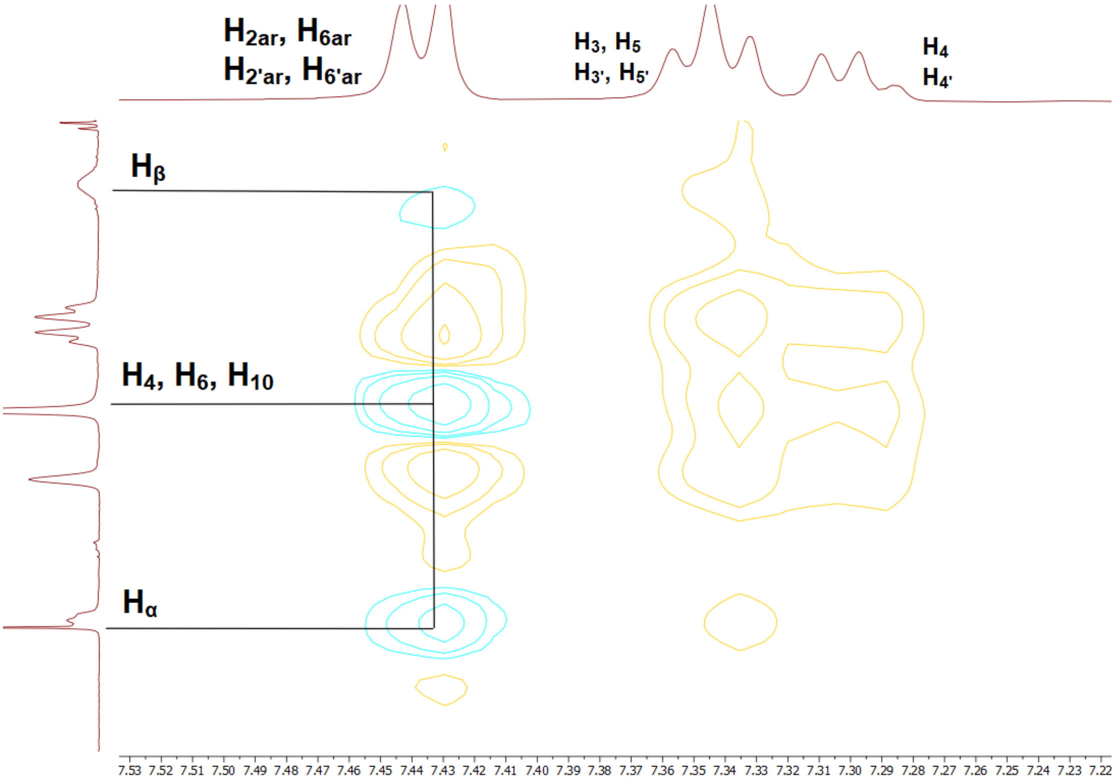

Figure S4. 2D NOESY of compound **I** at ambient temperature in D_2O.

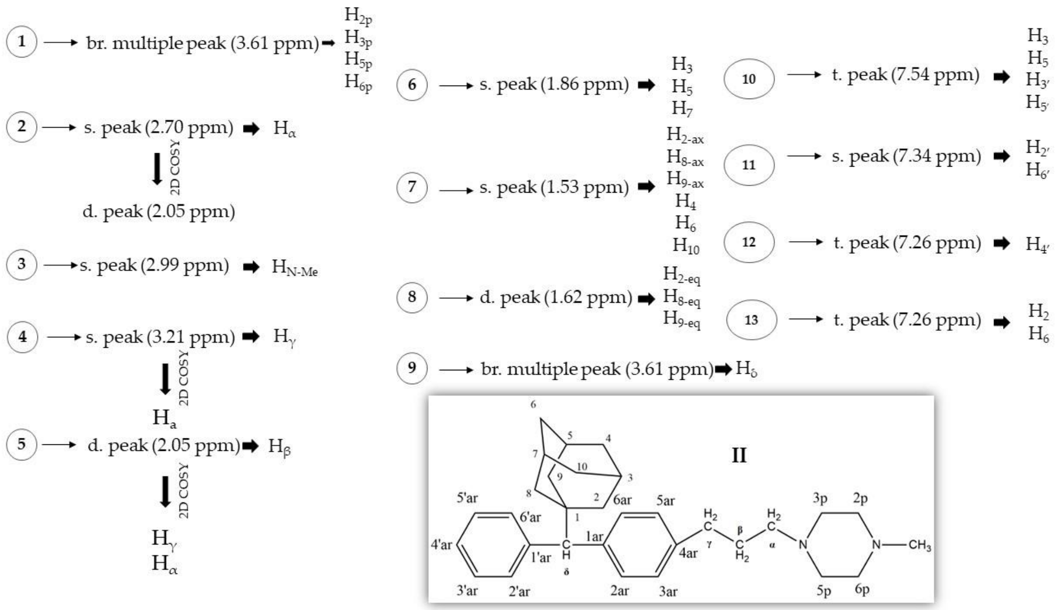

Figure S4. Molecular structure of compound **II** and the strategy followed.

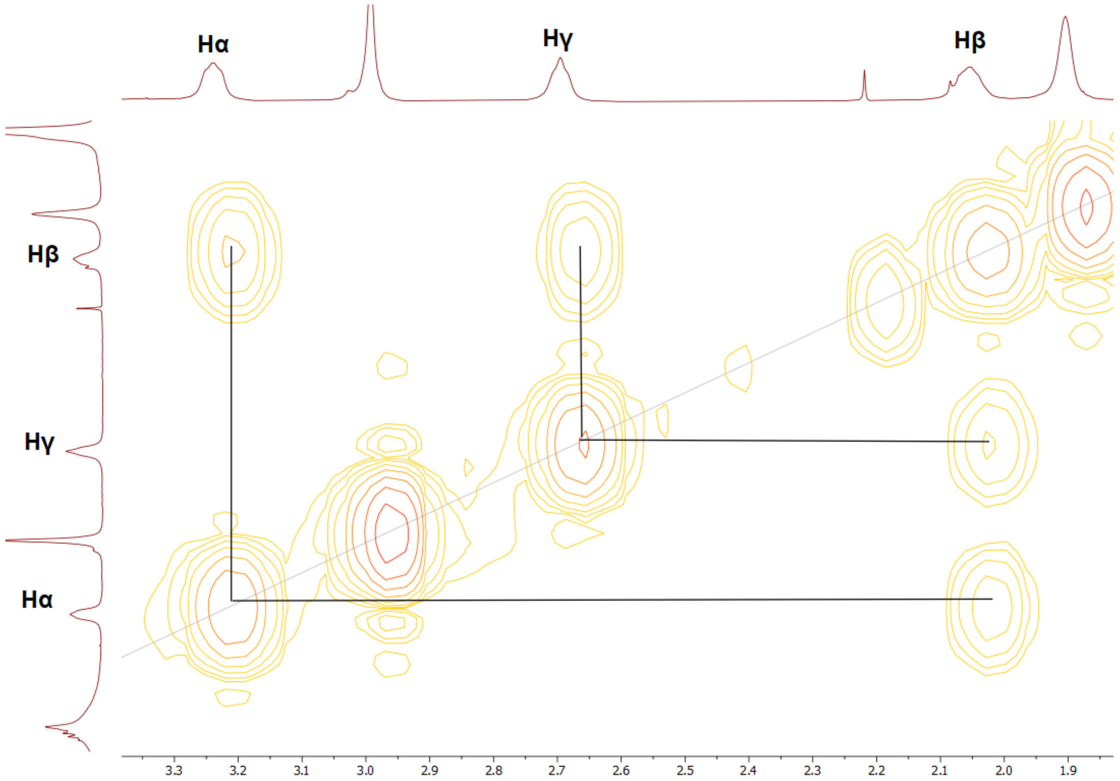

Figure S5: 2D COSY of compound **II** at ambient temperature in D_2O.

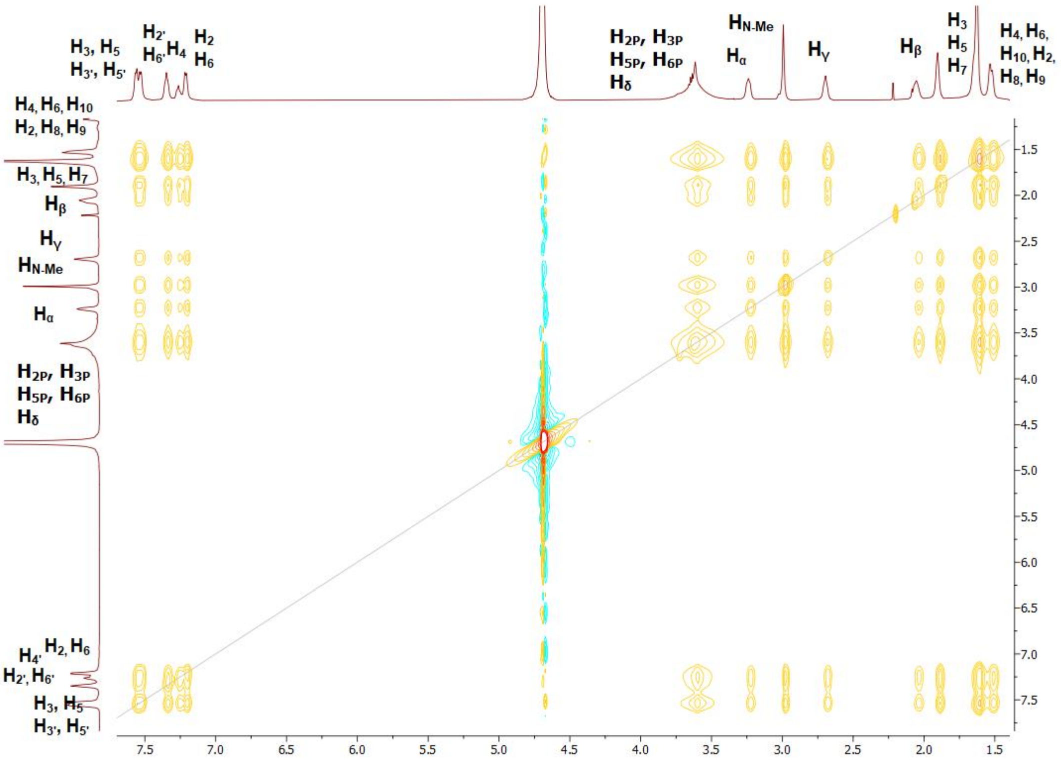

Figure S6: 2D NOESY of compound **II** at ambient temperature in D_2O.

Tables

Table S1. ^{1}H chemical structure shifts of compound **I** using a combination of 1D and 2D NMR experiments.

Position	^{1}H	Multiplicity	2D NOESY	2D COSY
**$H_α$**	2.69	d.		$H_β$ (1.30 ppm)
**$H_β$**	1.30	Br. S	H_{6ar}, H_{2ar}, $H_{2'ar}$, $H_{6'ar}$	
**$H_γ$**	2.21	Multiple	H_{6ar}, H_{2ar}, $H_{2'ar}$, $H_{6'ar}$	$H_β$ (1.30 ppm)
**H_2**	1.59	d.d.		
**H_3**	1.91	s.		
**H_4**	1.77	s.	H_{6ar}, H_{2ar}, $H_{2'ar}$, $H_{6'ar}$	
**H_5**	1.91	s.		
**H_6**	1.77	s.	H_{6ar}, H_{2ar}, $H_{2'ar}$, $H_{6'ar}$	
**H_7**	1.91	s.		
**H_8**	1.59	d.d.		
**H_9**	1.59	d.d.		
**H_{10}**	1.77	s.	H_{6ar}, H_{2ar}, $H_{2'ar}$, $H_{6'ar}$	
**H_{2ar}**	7.43	d.		
**H_{3ar}**	7.34	t.		
**H_{4ar}**	7.29	t.		
**H_{5ar}**	7.34	t.		
**H_{6ar}**	7.43	d.		
**$H_{2'ar}$**	7.43	d.		
**$H_{3'ar}$**	7.34	t.		
**$H_{4'ar}$**	7.29	t.		
**$H_{5'ar}$**	7.34	t.		
**$H_{6'ar}$**	7.43	d.		
**H_{2p}**	3.07	very br. s.		
**H_{3p}**	3.07	very br. s.		
**H_{5p}**	3.07	very br. s.		
**H_{6p}**	3.07	very br. s.		
**H_{N-Me}**	2.69	d.		$H_β$ (1.30 ppm)

Table S2. ^1H NMR chemical shifts of compound **II** derived from a combination of 1D and 2D homonuclear NMR experiments.

Position	^1H	Multiplicity	2D ^1H-^1H NOESY	2D ^1H-^1H COSY
H_α	2.70 ppm	s.	H_β<br>H_3, H_5, H_7<br>H_4, H_6, H_{10}<br>H_{2eq}, H_{8eq}, H_{9eq}	H_β (2.05)
H_β	2.05 ppm	d.	H_4, H_6, H_{10}<br>H_2, H_8, H_9	-
H_γ	3.21 ppm	d.	$H_{N\text{-}Me}$<br>H_α<br>H_β<br>H_3, H_5, H_7<br>H_4, H_6, H_{10}<br>H_2, H_8, H_9	H_β (2.05)
H_δ	3.61 ppm	br. multiplet		-
H_2	1.62 (eq) ppm<br>1.53 (ax) ppm	d.	H_3, H_5, H_7	-
H_3	1.86 ppm	s.	H_4, H_6, H_{10}<br>H_2, H_8, H_9	-
H_4	1.53 ppm	s.	H_3, H_5, H_7	-
H_5	1.86 ppm	s.	H_4, H_6, H_{10}<br>H_2, H_8, H_9	-
H_6	1.53 ppm	s.	H_3, H_5, H_7	-
H_7	1.86 ppm	s.	H_4, H_6, H_{10}<br>H_2, H_8, H_9	-
H_8	1.62 (eq) ppm<br>1.53 (ax) ppm	d.	H_3, H_5, H_7	-
H_9	1.62 (eq) ppm<br>1.53 (ax) ppm	d.	H_3, H_5, H_7	-
H_{10}	1.53 ppm	s.	H_3, H_5, H_7	-
H_{2ar}	7.22 ppm	d.		-
H_{3ar}	~7.54 ppm	t.	$H_{ar2'}$, $H_{ar6'}$<br>$H_{4ar'}$<br>H_{2ar}, H_{6ar}<br>H_3, H_5, H_7<br>H_4, H_6, H_{10}<br>H_2, H_8, H_9	-
H_{5ar}	~7.54 ppm	t.	$H_{2'ar}$, $H_{6'ar}$<br>$H_{4ar'}$<br>H_{2ar}, H_{6ar}<br>H_3, H_5, H_7<br>H_4, H_6, H_{10}<br>H_2, H_8, H_9	-
H_{6ar}	7.22 ppm	d.		-
$H_{2'ar}$	7.34 ppm	d.		-
$H_{3'ar}$	~7.54 ppm	t.	$H_{ar2'}$, $H_{ar6'}$<br>$H_{4ar'}$<br>H_{2ar}, H_{6ar}<br>H_3, H_5, H_7<br>H_4, H_6, H_{10}<br>H_2, H_8, H_9	-
$H_{4'ar}$	7.26 ppm	t.		-
$H_{5'ar}$	~7.54 ppm	t.	$H_{2'ar}$, $H_{6'ar}$<br>$H_{4'ar}$<br>H_{2ar}, H_{6ar}	-

			H_3, H_5, H_7<br>H_4, H_6, H_{10}<br>H_2, H_8, H_9	
**$H_{6'ar}$**	7.34 ppm	d.		-
**H_{2p}**	3.61 ppm	br. multiplet	H_α<br>$H_{N\text{-}Me}$<br>H_γ<br>H_β<br>H_3, H_5, H_7<br>H_4, H_6, H_{10}<br>H_2, H_8, H_9<br>H_{3ar}, H_{5ar},<br>$H_{3'ar}$, $H_{5'ar}$<br>H_{2ar}, H_{6ar}<br>H_{4ar}	-
**H_{3p}**	3.61 ppm	br. multiplet	H_α<br>$H_{N\text{-}Me}$<br>H_γ<br>H_β<br>H_3, H_5, H_7<br>H_4, H_6, H_{10}<br>H_2, H_8, H_9<br>H_{3ar}, H_{5ar},<br>$H_{ar3'}$, $H_{5'ar}$<br>H_{2ar}, H_{6ar}<br>H_{ar4}	-
**H_{5p}**	3.61 ppm	br. multiplet	H_α<br>$H_{N\text{-}Me}$<br>H_γ<br>H_β<br>H_3, H_5, H_7<br>H_4, H_6, H_{10}<br>H_2, H_8, H_9<br>H_{3ar}, H_{5ar},<br>$H_{3'ar}$, $H_{5'ar}$<br>H_{2ar}, H_{6ar}<br>H_{4ar}	-
**H_{6p}**	3.61 ppm	br. multiplet	H_α<br>$H_{N\text{-}Me}$<br>H_γ<br>H_β<br>H_3, H_5, H_7<br>H_4, H_6, H_{10}<br>H_2, H_8, H_9<br>H_{ar3}, H_{ar5},<br>$H_{ar3'}$, $H_{ar5'}$<br>H_{ar2}, H_{ar6}<br>H_{ar4}	-
**$H_{N\text{-}Me}$**	2.99 ppm	d.	H_α<br>H_β<br>H_γ<br>H_3, H_5, H_7<br>H_4, H_6, H_{10}<br>H_2, H_8, H_9<br>H_{2ar}, H_{6ar}<br>H_{3ar}, H_{5ar}<br>$H_{3'ar}$, $H_{5'ar}$	-
